# Presumed Chemotherapy-Induced Optic Neuropathy and Maculopathy: A Case Report

**DOI:** 10.2174/1874364101711010298

**Published:** 2017-09-30

**Authors:** David J. Mathew, Anupriya Arthur, Sheeja Susan John

**Affiliations:** Department of Ophthalmology, Christian Medical College, Vellore 632001, Tamil Nadu, South India

**Keywords:** Cancer chemotherapy, Toxic optic neuropathy, Toxic maculopathy, Cytarabine, Daunorubicin, AML

## Abstract

**Purpose:**

With the advent of more aggressive cytotoxic chemotherapy regimens, the incidence of ocular toxicity due to these drugs is also on the rise. We report a case of Presumed Chemotherapy-Induced optic neuropathy and maculopathy secondary to treatment with cytarabine and daunorubicin for Acute Myeloid Leukaemia (AML).

**Case report:**

A 50-year-old man with AML developed sudden decrease in vision in his left eye after three cycles of chemotherapy with cytarabine and daunorubicin. He presented to us six weeks later with bilateral optic atrophy and foveal atrophic changes with early bull’s eye maculopathy. A diagnosis of presumed chemotherapy-induced optic neuropathy with maculopathy was made, and the patient was put on an alternative chemotherapeutic regimen. There was no further decrease in vision on follow up.

**Conclusion:**

To the best of our knowledge, this is the first report of clinically demonstrable macular toxicity in the form of macular atrophic changes and bull’s eye maculopathy associated with the use of cytarabine and daunorubicin. Early diagnosis and appropriate management of such cases is imperative to prevent further visual deterioration.

## 1 INTRODUCTION

The incidence of cytotoxic chemotherapy-related ocular toxicity is on the rise with the advent of newer agents and more aggressive combination regimens [1]. It is often difficult to arrive at a specific cause-effect relationship between the use of chemotherapeutic drugs and their toxic effects in such cases. The use of multiple drugs for chemotherapy, and the combination of radiotherapy and chemotherapy make interpretation of the probable toxicity of these drugs difficult. Patients on chemotherapy are also often not able to cooperate fully for extensive investigations and follow up regimes for the ocular condition due to their poor general health, and the management of the systemic disease takes priority in many of these patients. The quality of life of the patient is further compromised due to poor vision in such cases. We report a case of presumed chemotherapy-induced optic neuropathy and maculopathy associated with the use of cytarabine and daunorubicin for Acute Myeloid Leukaemia (AML).

## 2 CASE REPORT

A 50-year-old man, who was diagnosed with Acute Myeloid Leukemia (AML M1), developed sudden decrease in vision in his left eye after three cycles of intravenous cytarabine and daunorubicin. An MRI scan done two days after the onset of the symptoms was unremarkable. As his general condition was poor, further investigations and treatment were deferred at this point. He was started on oral prednisolone at the dose of 1mg/kg/day three weeks after the onset of decreased vision. Prednisolone was subsequently tapered and stopped, and he was referred to our centre for further management.

**Fig. (1):**
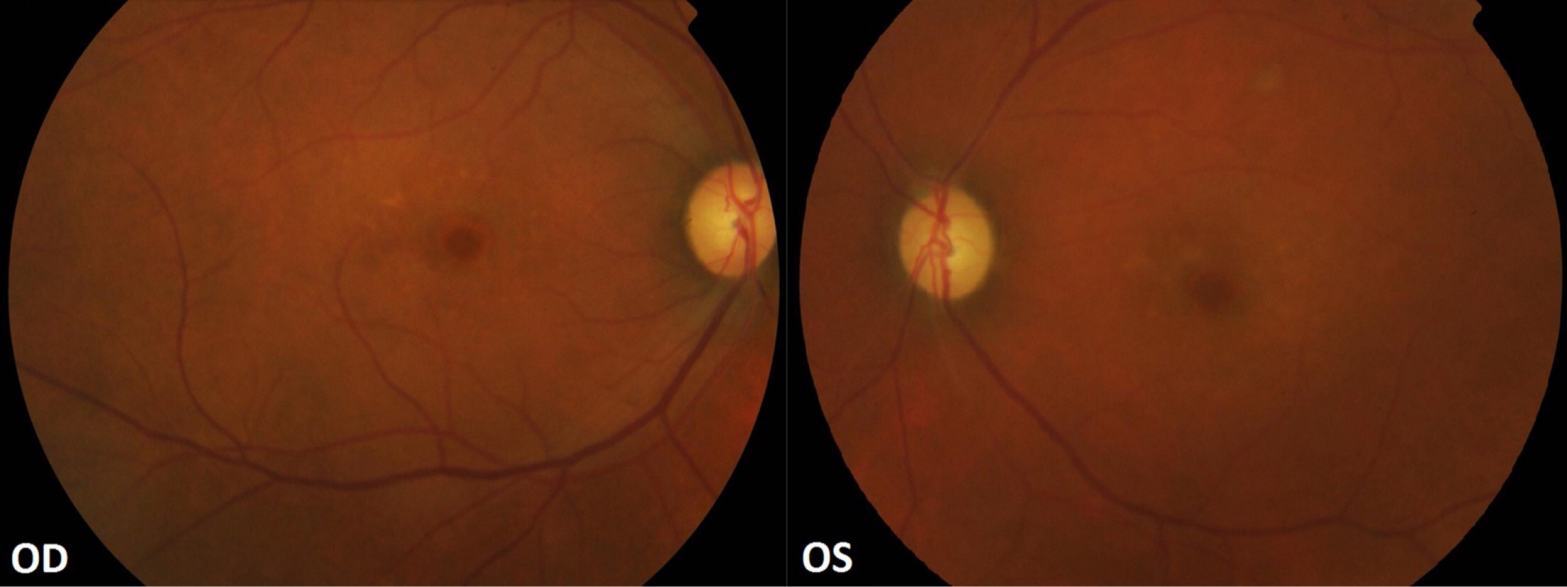
Color fundus photograph showing optic disc pallor and foveal atrophic changes in a bull’s eye configuration in both eyes.

He presented at our centre, six weeks after the onset of symptoms, with a best corrected visual acuity of 20/20 in the right eye and 20/30 in the left eye. Colour vision, tested using Ishihara pseudo-isochromatic plates, was defective in both eyes. He had a relative afferent pupillary defect in his left eye. The intra ocular pressure was normal in both eyes. The vitreous was clear; there was pallor of both optic discs with a cup-to-disc ratio of 0.3. Foveal atrophic changes in an early ‘bull’s eye’ pattern were noted in both eyes (Fig. **1**).

**Fig. (2):**
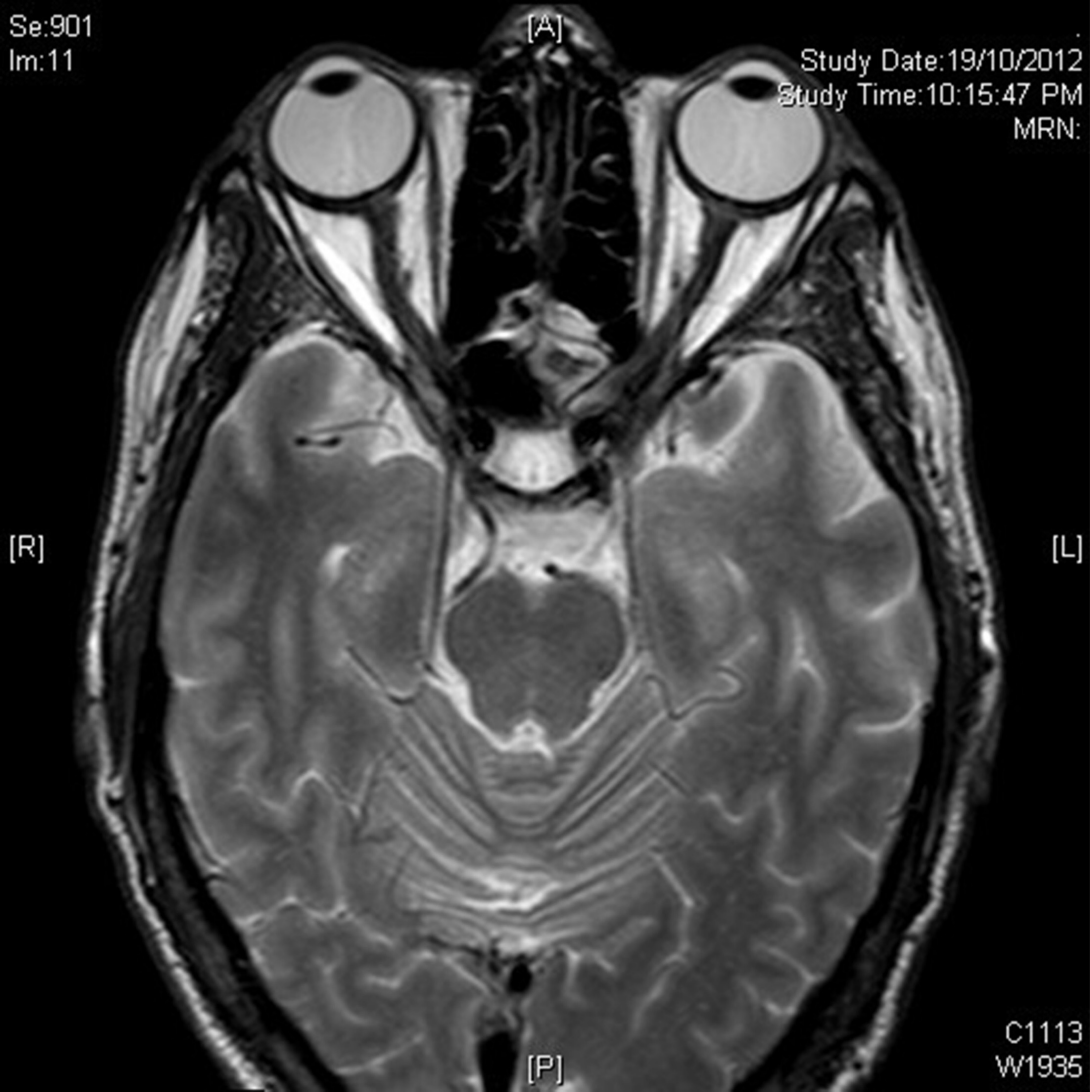
MRI scan of the orbit showing no evidence of active neuritis or infiltration of the optic nerves.

**Fig. (3):**
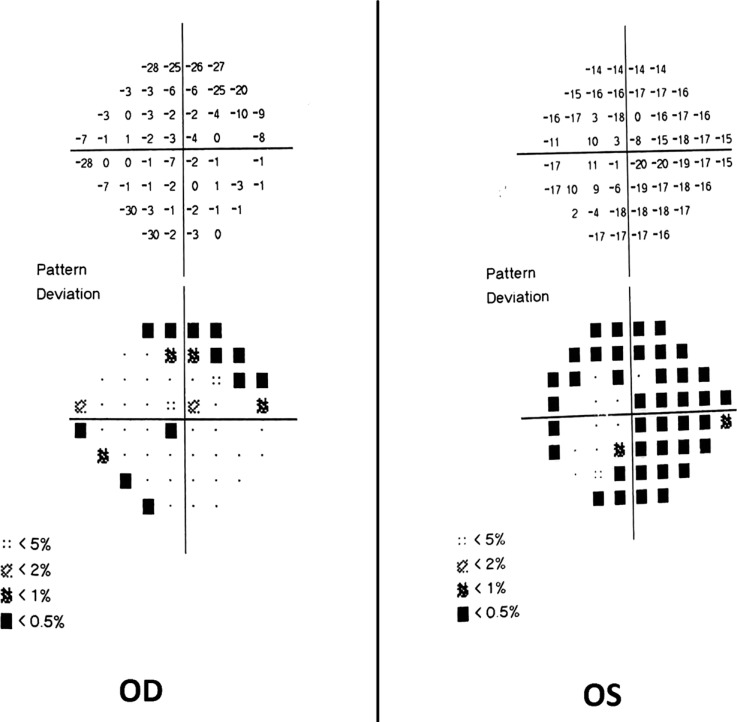
Automated perimetry showing peripheral depressed points in the right eye and constriction of the visual field in the left eye with a small temporal island of vision.

**Fig. (4):**
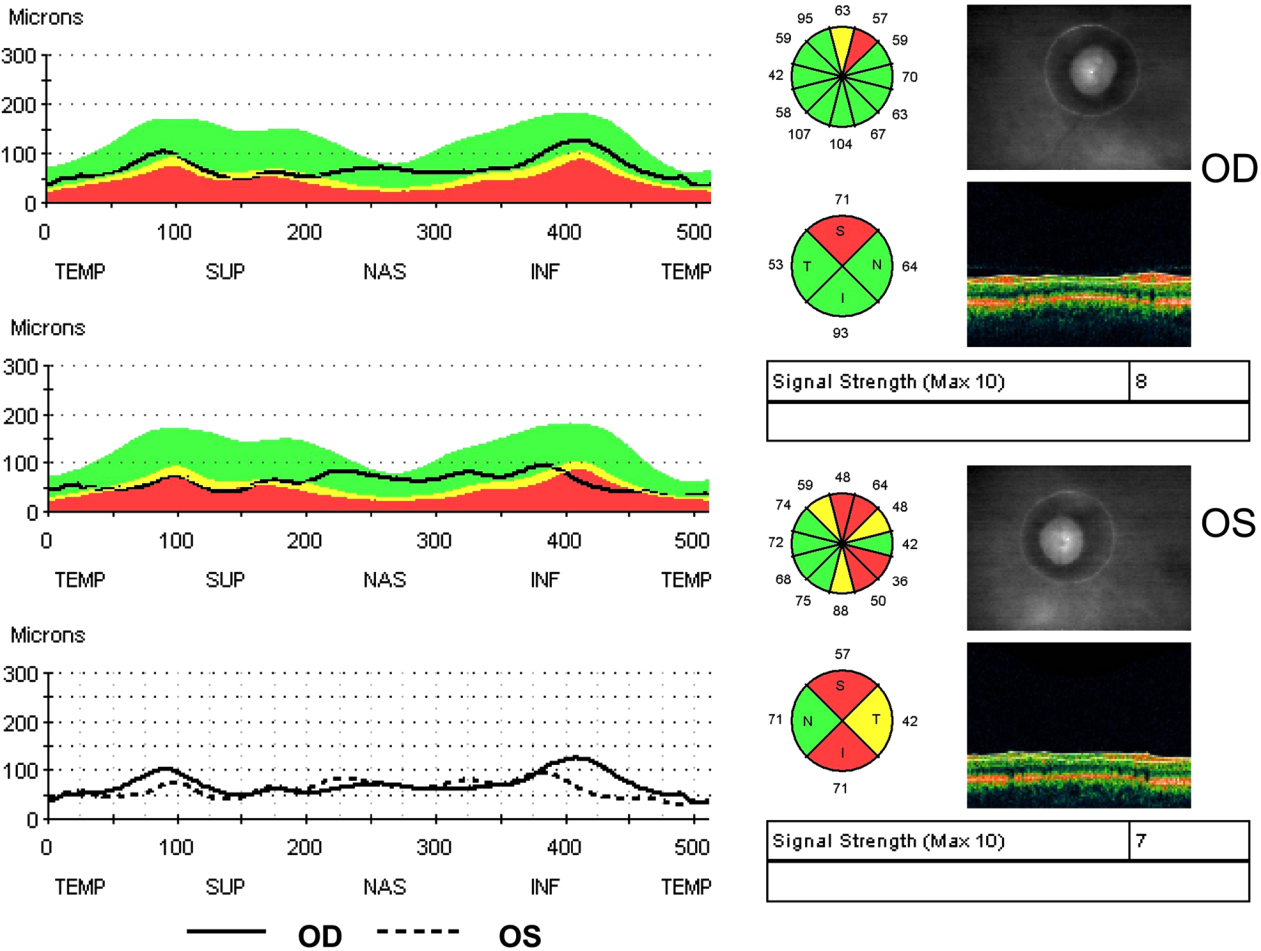
Optical coherence tomography (OCT) showing thinning of the retinal nerve fibre layer in both eyes, left eye being more affected than the right.

**Fig. (5):**
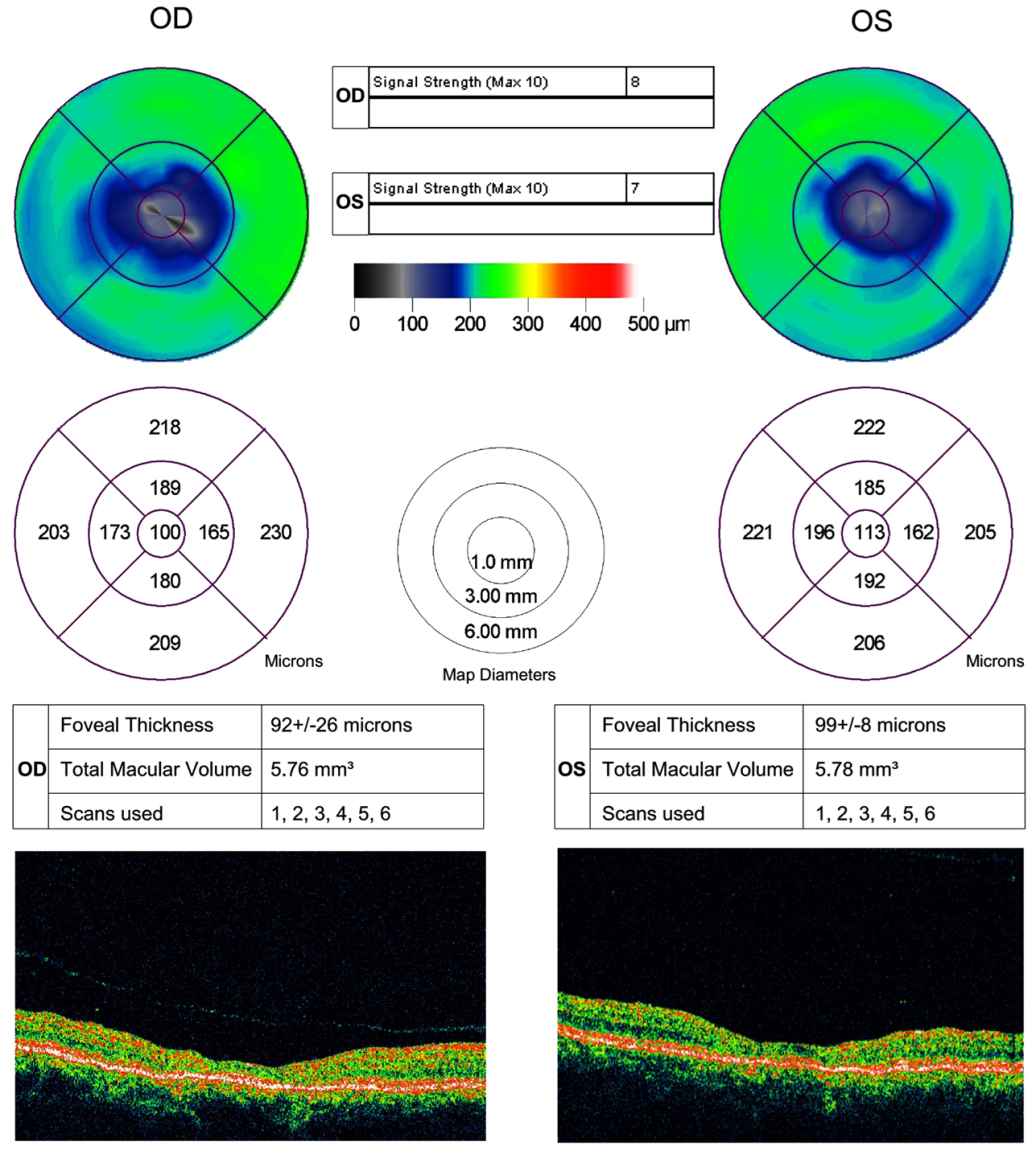
OCT of the macula showing thinning and alteration of the foveal contour.

**Fig. (6):**
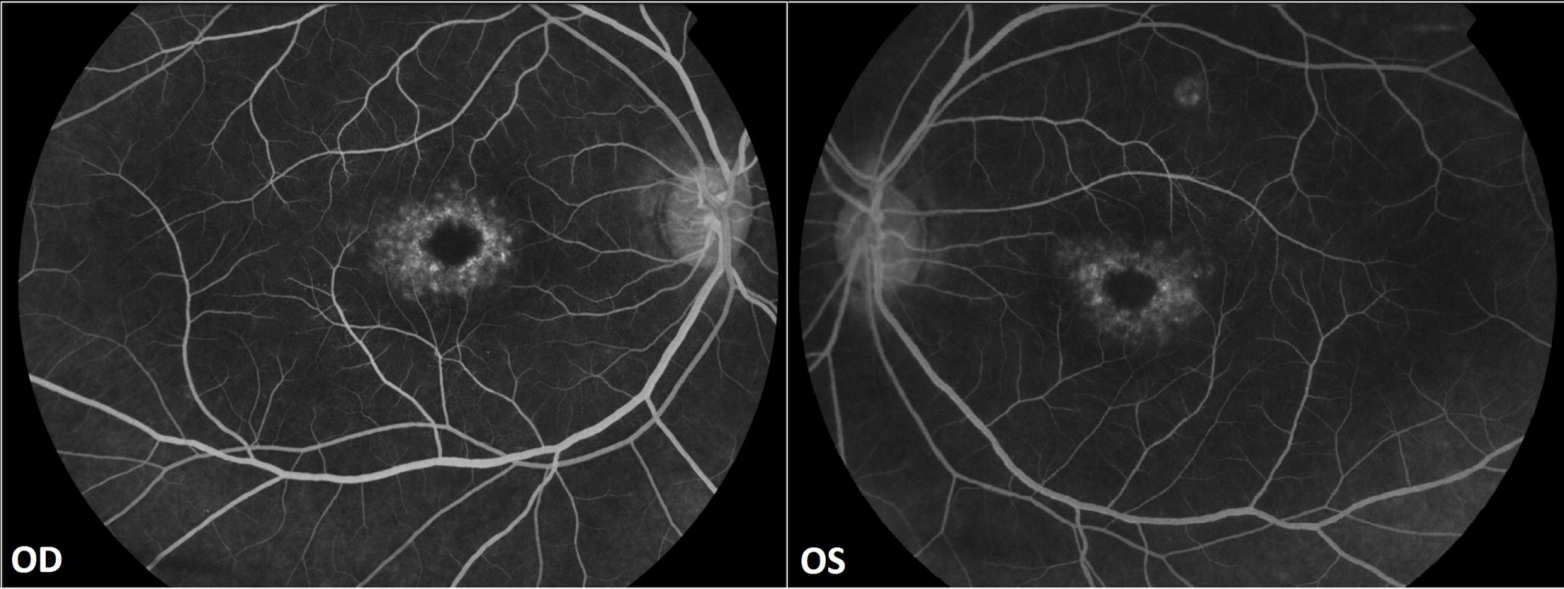
Fundus fluorescein angiography showing window defects with mottled hyperfluorescence in the parafoveal region in both eyes.

No evidence of active neuritis or infiltration of the optic nerves was found on review of the MRI scan of the orbits (Fig. **2**). Automated perimetry revealed peripheral depressed points in the right eye and constriction of the visual field in the left eye with a small temporal island of vision (Fig. **3**). Optical coherence tomography (OCT) revealed thinning of the retinal nerve fibre layer in both eyes (left eye > right eye) (Fig. **4**). OCT of the macula confirmed thinning and alteration of the foveal contour in both eyes (Fig. **5**). Fundus fluorescein angiography showed window defects with mottled hyperfluorescence in the parafoveal region in both eyes. Late staining of the disc was noted in both eyes with no leak at the macula or disc (Fig. **6**). Electrophysiological tests were not performed as these were not available at our centre.

A diagnosis of presumed chemotherapy-induced optic neuropathy with maculopathy was made. The patient underwent another cycle of chemotherapy with a different set of drugs, followed by bone marrow transplantation. There was no further deterioration of vision, or progression of optic nerve and macular changes as documented by fundus photography, OCT and automated perimetry, in either eye over a six-month follow-up period.

## 3 DISCUSSION

In the present era, while the advances in the field of cancer chemotherapy have been a boon to many patients suffering from various forms of malignancy, there has been a concomitant rise in the reports of newer, hitherto unheard of, adverse effects of these drugs [1]. It is often difficult to establish a specific cause-effect relationship between the use of cytotoxic chemotherapeutic drugs and complications related to their toxicity. The interpretation of the possible toxic effects of these drugs is not easy as most patients are on multiple chemotherapeutic drugs, or on radiotherapy in addition to chemotherapy. Moreover, ophthalmic manifestations may be part of the disease process. Retinal and vitreous hemorrhages, cotton wool spots, retinal venous occlusions, and leukemic infiltrates of the retina, choroid and orbits with proptosis and exudative retinal detachment, have been described in acute myeloid leukemia [2, 3].

The differentials of optic neuropathy in our patient would include toxic, infiltrative, compressive and demyelinating optic neuropathy. Imaging ruled out the latter three causes in this case.

The differentials of maculopathy in this patient include Heredomacular dystrophy (Progressive cone dystrophy, benign concentric annular macular dystrophy) and drug toxicity. A diagnosis of Heredomacular dystrophy is unlikely in this patient as the onset of the disease is usually at a younger age, and it is associated with photophobia, nystagmus and poor vision in most cases. Moreover, the onset of symptoms usually precedes the onset of signs. The patient was not on any drugs like chloroquine, hydoxychloroquine, desferrioxamine and methotrexate, which are known to cause maculopathy [4].

There have been a few reports of toxic optic neuropathy associated with both cytarabine and daunorubicin [5, 6]. Other ocular toxic effects related to the use of cytarabine include corneal epithelial toxicity and hemorrhagic conjunctivitis [6].

There have been no reports of retinal toxicity specifically associated with the use of cytarabine till date. However, in seven patients who received cytarabine in combination with whole body irradiation and intrathecal methotrexate, Vogler et al [7] noted microvascular changes including cotton wool spots, capillary nonperfusion, macular edema and neovascularisation. The microvascular changes noted are, however, highly suggestive of radiation retinopathy. Methotrexate has also been noted to cause macular edema [8]. Hence, the retinal changes in these cases cannot be attributed to cytarabine alone.

Daunorubicin has been reported to cause retinal toxicity with high intraocular doses in experimental settings [9]. There has also been a report of acute reversible maculopathy in a patient treated with daunorubicin in combination with desferrioxamine and iron sorbitol citrate [4]. However, there have been other reports of maculopathy attributable to desferrioxamine [10, 11]. Therefore, as with Vogler et al’s case series, a specific association between the use of daunorubicin and the occurrence of maculopathy cannot be elucidated in the above case. To the best of our knowledge, there have been no reports of macular thinning or bull’s eye maculopathy, as seen in our case, associated with the clinical use of these drugs for chemotherapy.

Daunorubicin has been considered for ocular use to inhibit proliferative vitreoretinopathy changes after surgery for retinal detachment [12, 13], and as an adjunct in glaucoma filtering surgeries [14] due to its anti fibroblast action. Hence, it is of paramount importance to further investigate the possibility of occurrence of macular toxicity with the clinical use of this drug.

## CONCLUSION

With the increasing use of more aggressive chemotherapeutic regimens for leukemia, reports of hitherto unreported drug toxicity are emerging. Optic nerve toxicity associated with cytarabine and daunorubicin has been reported in rare instances [5, 6]. Daunorubicin has been reported to cause retinal toxicity with high intraocular doses in experimental settings [9]. However, to the best of our knowledge, this is the first report of probable macular toxicity in the form of macular thinning and bull’s eye maculopathy associated with the clinical use of these drugs for chemotherapy.

It is often difficult to arrive at a specific cause- effect relationship between chemotherapeutic drugs and their toxic effects due to the use of multiple drugs for chemotherapy, the combination of radiotherapy and chemotherapy, as well as the difficulties with appropriate documentation of the ocular complications as these patients are often not able to cooperate fully for extensive investigations and follow up regimes for the ocular condition due to their poor general health.

Ocular complications secondary to cytotoxic chemotherapy are often neglected as in this case, since the systemic disease takes priority. Early detection, accurate documentation and diagnosis, and appropriate management can help reduce the severity of toxic effects and limit permanent structural damage and functional disability.

A baseline ophthalmic examination of patients before the initiation of chemotherapeutic regimens, and regular follow-up thereafter, coupled with education of the patient regarding possible drug toxicity, can go a long way in diagnosing the toxic effects of these drugs at an early stage. Oncologists and Ophthalmologists should work together to facilitate early referral and prevention of irreversible ocular toxicity due to chemotherapeutic drugs.
